# Supplementary material for: Psychometric validation and cultural adaptation of executive functioning scale in Malaysian University students
Source: PLoS One. 2026 Apr 3;21(4):e0341453. doi: 10.1371/journal.pone.0341453 (PMC13048388; doi:10.1371/journal.pone.0341453)
Supplement: S2 File — The English version of the scale, having undergone localised validity and reliability testing, is detailed in this document. (DOCX) [file pone.0341453.s002.docx]

Dear Participant,

Thank you for participating in this research initiative. Please carefully review these guidelines before proceeding:

Purpose: This standardized assessment measures behavioral frequencies and emotional experiences over the past 14 days.

Recall Process: Take a moment to mentally reconstruct your daily routines and significant events from the last two weeks.

Response Method: After reading each statement, select the numerical rating that most accurately reflects your experience frequency.

Scale Reference:
0 = Never
1 = Rarely
2 = Sometimes
3 = Often
4 = Very often

Confidentiality Assurance: All responses are anonymized and stored securely for academic analysis.

Please provide honest responses reflecting your genuine experiences. Your thoughtful participation contributes significantly to scientific understanding.

| No | Item | Options | | | | |
| --- | --- | --- | --- | --- | --- | --- |
| 1 | How quickly do you think you can understand and execute a simple instruction, such as "Please sit down"? | 0 | 1 | 2 | 3 | 4 |
| 2 | How quickly do you think you can understand and execute an instruction with two steps, such as "Take out your textbook, then open it to page 10"? | 0 | 1 | 2 | 3 | 4 |
| 3 | How quickly do you think you can understand and execute an instruction with several steps, such as dribbling, passing, and shooting in basketball? | 0 | 1 | 2 | 3 | 4 |
| 4 | I can quickly master a series of newly learned actions. | 0 | 1 | 2 | 3 | 4 |
| 5 | I am good at remembering the sequence of events. | 0 | 1 | 2 | 3 | 4 |
| 6 | I can follow simple instructions well. | 0 | 1 | 2 | 3 | 4 |
| 7 | I am skilled at following very complex instructions (e.g., I am effective at following a multi-stage research protocol or laboratory procedure). | 0 | 1 | 2 | 3 | 4 |
| 8 | I tend to be reckless in pursuit of what I want. | 0 | 1 | 2 | 3 | 4 |
| 9 | I crave new and exciting experiences. | 0 | 1 | 2 | 3 | 4 |
| 10 | I don't consider potential dangers much when doing things. | 0 | 1 | 2 | 3 | 4 |
| 11 | I tend to act impulsively. | 0 | 1 | 2 | 3 | 4 |
| 12 | I am willing to take risks that most people wouldn't dare. | 0 | 1 | 2 | 3 | 4 |
| 14 | I get frustrated easily. | 0 | 1 | 2 | 3 | 4 |
| 15 | I find it hard to comfort myself in difficult situations | 0 | 1 | 2 | 3 | 4 |
| 16 | I feel defeated easily when facing difficulties | 0 | 1 | 2 | 3 | 4 |
| 17 | I can calm down quickly after being excited | 0 | 1 | 2 | 3 | 4 |
| 18 | I find it hard to lift my spirits in adversity | 0 | 1 | 2 | 3 | 4 |
| 19 | I immerse myself in sad or exciting emotions more easily than others | 0 | 1 | 2 | 3 | 4 |
| 20 | It's hard for me not to show strong negative or positive emotions | 0 | 1 | 2 | 3 | 4 |
| 21 | I am easily agitated | 0 | 1 | 2 | 3 | 4 |
| 22 | I find it difficult to manage my emotions | 0 | 1 | 2 | 3 | 4 |
| 23 | I can distract myself from unpleasant events | 0 | 1 | 2 | 3 | 4 |
| 24 | I can regain control of my emotions after feeling upset | 0 | 1 | 2 | 3 | 4 |
| 25 | I seem to process information slowly | 0 | 1 | 2 | 3 | 4 |
| 26 | I respond slowly even when doing things I enjoy | 0 | 1 | 2 | 3 | 4 |
| 27 | I work quickly and accurately in activities | 0 | 1 | 2 | 3 | 4 |
| 28 | I do things slowly even when interested | 0 | 1 | 2 | 3 | 4 |
| 29 | I think before I act or speak | 0 | 1 | 2 | 3 | 4 |
| 30 | I can suppress certain behaviors when asked | 0 | 1 | 2 | 3 | 4 |
| 31 | I stop what I'm doing when asked to stop | 0 | 1 | 2 | 3 | 4 |
| 32 | I am willing to wait to get what I want | 0 | 1 | 2 | 3 | 4 |
| 33 | I can stop doing what I like when I have to complete other tasks | 0 | 1 | 2 | 3 | 4 |
| 34 | I can quickly get back into the state even after being interrupted | 0 | 1 | 2 | 3 | 4 |
| 36 | I can transition freely between different tasks with ease | 0 | 1 | 2 | 3 | 4 |
| 37 | I miss important information because I focus on what I'm doing | 0 | 1 | 2 | 3 | 4 |
| 38 | I think carefully about the consequences before acting | 0 | 1 | 2 | 3 | 4 |
| 39 | I can resist immediate desires that are not good in the long run | 0 | 1 | 2 | 3 | 4 |
| 40 | I can focus on completing important tasks without being distracted by more interesting activities | 0 | 1 | 2 | 3 | 4 |
| 41 | I can change my behavior based on feedback from others | 0 | 1 | 2 | 3 | 4 |
| 42 | I can closely imitate others' behaviors and actions | 0 | 1 | 2 | 3 | 4 |
| 43 | I can remember multiple pieces of information or things simultaneously | 0 | 1 | 2 | 3 | 4 |
| 44 | I find it hard to handle multiple things in my mind simultaneously | 0 | 1 | 2 | 3 | 4 |
| 45 | I am good at mentally organizing information | 0 | 1 | 2 | 3 | 4 |
| 46 | I am good at remembering how things happened exactly | 0 | 1 | 2 | 3 | 4 |
| 47 | I can easily transition from one activity to another | 0 | 1 | 2 | 3 | 4 |
| 48 | I find it hard to stop what I'm doing and start something new | 0 | 1 | 2 | 3 | 4 |
| 49 | I can easily switch back and forth between two activities that require attention | 0 | 1 | 2 | 3 | 4 |
| 50 | I can persist in doing something continuously | 0 | 1 | 2 | 3 | 4 |
| 51 | I feel overwhelmed when I have to do many things at once | 0 | 1 | 2 | 3 | 4 |
| 52 | I can stop doing something I am interested in to do what I need to do | 0 | 1 | 2 | 3 | 4 |

***Note.*** The localised adaptation of this version retains the original item numbers while making appropriate content adjustments. During formal administration, the omitted Item 13 and Item 35 may be designated by the user as screening items to filter out invalid scales.

The items corresponding to each dimension in this scale are as follows:

| Constructs | Items |
| --- | --- |
| Working Memory | 1 - 7, 42 - 46 |
| Risk Avoidance | 8 - 12, 21, 38 |
| Response Inhibition | 29 - 33, 39 - 41 |
| Emotional Regulation | 14 - 20, 22 - 24 |
| Set Shifting | 34, 36, 37, 47 - 52 |
| Processing Speed | 25 -28 |
| Overall EF | Sum |
