# Supplementary material for: Psychometric validation and cultural adaptation of executive functioning scale in Malaysian University students
Source: PLoS One. 2026 Apr 3;21(4):e0341453. doi: 10.1371/journal.pone.0341453 (PMC13048388; doi:10.1371/journal.pone.0341453)
Supplement: Attachment — Submitted filename: Response to Reviewers.docx [file pone.0341453.s004.docx]

****Response to Reviewers****

Dear Editor and Reviewers,
Thank you for the opportunity to revise our manuscript, "Psychometric Validation and Cultural Adaptation of the Executive Functioning Scale in Malaysian University Students" (MS ID: [PONE-D-25-59203]). We are deeply grateful to you and the esteemed reviewers for providing such insightful and constructive feedback. The comments have been invaluable in helping us to strengthen the manuscript. We have addressed all points raised by the reviewers and detail these revisions below in a point-by-point response.

*****Response to Editor*****

Regarding the requested citations for Table 1 and Figure 3 in the main text, we have highlighted these in red within the document titled "Revised Article with Changes Highlighted", on pages 7 and 15 respectively. These were correctly cited in the original version. Please refer to the red highlights on these two pages within that document. Thank you.

*****Response to Reviewer #1*****

We sincerely thank Reviewer #1 for their comprehensive and thoughtful review. We particularly appreciate his emphasis on the critical issue of cultural bias in cognitive assessment, which has been instrumental in enhancing the theoretical framing and practical implications of our study.

****1. Comment on Developing the Issue of Cultural Bias, Its Impact, and Implications for Equity:****
The reviewer correctly pointed out that the manuscript could more thoroughly develop the problem of cultural bias inherent in Western-developed tools, its downstream impact on academic and mental health support, and its broader implications for equity in higher education.

**Response**: We are grateful for this critical suggestion. To more robustly frame the rationale for our study, we have incorporated a new, comprehensive paragraph into the Introduction section (now the second paragraph). This section now explicitly discusses the WEIRD-centric origins of most EF tools, the concept of cultural dissonance, the risk of generating "false deficits," and the profound consequences of biased assessment for educational equity and inclusivity, thereby directly addressing the reviewer's primary concern.

****2. Comment on Key Strategies for Improving Transcultural Validity (Points a, b, c, d):****
The reviewer outlined a set of key best-practice strategies for cross-cultural adaptation, including a) ensuring linguistic and conceptual equivalence, b) adapting scenarios for cultural relevance, c) conducting rigorous psychometric validation, and d) incorporating local norms and expert input.

**Response**: We thank the reviewer for articulating these essential methodological standards. We are pleased to confirm that our study's methodology did, in fact, incorporate these steps. To make this explicit and to enhance methodological transparency, we have added a new, dedicated paragraph within the Methods section. This new section now systematically details our procedures, framing them using the reviewer's excellent recommendations:

**Regarding points (a) & (b)**: We now explicitly describe our use of a standardized forward-backward translation protocol to establish linguistic equivalence and provide concrete examples (e.g., revising the "baking" item to a "research protocol" scenario) to illustrate how we ensured conceptual and contextual equivalence.

**Regarding point (c)**: We reinforce that the purpose of the sequential EFA and CFA, detailed in the "Construct Validity" subsection, was precisely to achieve psychometric validation and confirm the scale's structural integrity within the Malaysian context.

**Regarding point (d)**: We have clarified that an expert panel, comprising two local educational psychologists and one linguist, was engaged to review the adapted items to ensure alignment with local norms and cultural appropriateness.

****3. Comment on a Mixed-Methods Approach (Point e):****
The reviewer astutely suggested that a mixed-methods approach would be a valuable direction for future research.

**Response**: This is an excellent suggestion for deepening the field's understanding. As our current study's scope was focused on the quantitative psychometric validation of the EFS, we have incorporated this recommendation into our manuscript. Specifically, we have added a new paragraph to the Limitations section acknowledging the study's reliance on quantitative methods and explicitly proposing a mixed-methods design as a critical pathway for future research to explore the nuanced cultural interpretations of executive functioning.

*****Response to Reviewer #2*****

We are greatly encouraged by Reviewer #2’s highly positive assessment of our manuscript. We thank reviewer for his supportive comments on the study's rigor and for his helpful and constructive suggestions for minor revisions.

****1. Comment on the Cultural Adaptation Process:****
The reviewer requested clarification on the number of experts involved in the review panel and the process by which consensus was achieved.

**Response**: We thank the reviewer for this important point on methodological transparency. We have revised the new "Cultural Adaptation of the Executive Functioning Scale" subsection within the Methodology. The text now clearly states that the expert panel comprised "two local educational psychologists and one linguist." We have also added that consensus regarding item wording and cultural appropriateness was reached through iterative, consensus-based discussion among the panel members and the research team.

****2. Comment on the Presentation of Table 3:****
The reviewer noted that the presentation of Table 3 could be improved for clarity and interpretability.

**Response**: We are grateful for this feedback on data presentation. To enhance the clarity and interpretability of Table 3, we have reformatted it. The revised table now presents only the item numbers corresponding to each factor, rather than the full item text. This significantly improves readability and aligns with standard reporting practices for factor analysis results.

We are confident that these revisions have substantially improved the manuscript and thoughtfully address all the concerns raised. We look forward to your and the reviewers' positive evaluation of the revised submission.

Sincerely,
All authors.
